# Acquired Labial Adhesion in a Reproductive-Aged woman secondary to Systemic Lupus Erythematosus

**DOI:** 10.12669/pjms.342.14364

**Published:** 2018

**Authors:** Dogukan Yildirim, Merve Talmac

**Affiliations:** 1 Dogukan Yildirim, Dogukan Yildirim, Merve Talmac, Department of Obstetrics and Gynecology, Kanuni Sultan Suleyman Training and Research Hospital, Kucukcekmece, Istanbul, Turkey; 2 Merve Talmac, Dogukan Yildirim, Merve Talmac, Department of Obstetrics and Gynecology, Kanuni Sultan Suleyman Training and Research Hospital, Kucukcekmece, Istanbul, Turkey

**Keywords:** Labial adhesion, Labial fusion, Systemic lupus erythematosus

## Abstract

Labial adhesion is a rare condition in reproductive-aged women. There are only a few reported cases of labial adhesion in this period of woman’s life. We herein, present a case of a 22-year-old G1P1 woman with a thick and fibrous labial adhesion. The labial adhesion was excised, and the labial mucosa was sutured under local anaesthesia. Her hormonal profile (FSH, LH and estrogen levels) was found to be normal. The patient was later diagnosed with systemic lupus erythematosus (SLE) by a rheumatologist. It is the first case report that shows a relationship between SLE and labial adhesion.

## INTRODUCTION

Puberty and menopause define the start and the end of the female reproductive life cycle. Prepubertal girls and postmenopausal women tend to have labial adhesions as a result of low estrogen levels. In contrast, labial adhesions are rarely seen in reproductive period. There are a few reports in reproductive age women related to female circumcision, lichen sclerosis, herpes simplex, diabetes, pemphigoid, and caustic vaginitis.^1^

## CASE REPORT

**Fig.1:**
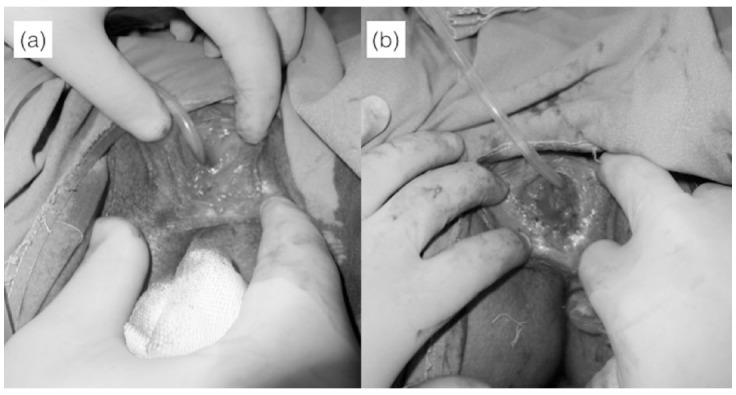
(a) Preoperative view of the thick fibrous labial adhesion (b) Early postoperative view after excising the adhesion.

A 22-year-old G1P1 woman presented to the outpatient gynecology clinic of the Kanuni Sultan Suleyman Training and Research Hospital in Istanbul with the complaint of inability to have sexual intercourse with her husband during the last year. She had a history of preeclampsia followed by a spontaneous vaginal delivery and an eclamptic seizure in the postpartum period three years previously. In the first two years following delivery, she reported to have no problems during sexual intercourse. On the third year, however, her husband started having difficulty inserting his penis into her vagina. During the same period, she had regular menses and had no complaints of vaginal discharge, urinary symptoms or a history of pelvic trauma. Upon gynecological examination, a thick and fibrous labial adhesion was detected (Fig.1). A 1cm opening was observed between the external urethral meatus and the labial adhesion. Her hormonal profile (FSH, LH and estrogen levels) was found to be normal. The labial adhesion was excised, and the labial mucosa was sutured under local anaesthesia. The patient was discharged from the hospital, and a topical antibacterial cream was prescribed twice daily for one week. She was instructed to abstain from sexual intercourse for four weeks after the surgery. On the twelfth postoperative week during follow-up, she reported no difficulties during sexual intercourse. However, she complained of a painless oral ulcerous lesion.

A more detailed history revealed that oral ulcers had been developing on a recurring basis in the past. She further reported a painless vaginal ulcer which was spontaneously healed in two weeks before experiencing difficulties in sexual intercourse. Considering also the poor obstetric history of the patient, we had brought suspicion upon a connective tissue disorder. The patient was referred to a rheumatologist for a specific evaluation. The immunological panel showed high anti-nuclear antibody (ANA), anti-double-stranded DNA (anti-dsDNA) antibody and anti-Smith (anti-Sm) antibody titers, with low complement three levels. Lupus anticoagulant and anticardiolipin IgG and IgM antibodies were negative. She also declared suffering from skin rashes on her hands developing upon exposure to sunlight. The patient was eventually diagnosed with systemic lupus erythematosus (SLE).

## DISCUSSION

Labial adhesion occurs when the labia minora adhere together resulting in a narrowed vaginal opening or introitus. It is most commonly seen in prepubertal girls and postmenopausal women; however, it is an extremely rare condition in women of the reproductive age group.^2^ The incidence is estimated to be 0.6-5% in prepubertal girls.^3^ The diagnosis is made by the inspection of the vulva. The adhesion typically begins at the posterior fourchette and continues anteriorly.^4^ Though it is often an acquired condition, it can also occur as a congenital anomaly, usually accompanied by other genitourinary abnormalities.^2^ It can be asymptomatic, especially in prepubertal girls. Anterior adhesions may lead to urinary symptoms such as urinary retention, urinary tract infection, pain or altered urinary stream.^2^ The most common complaint in women of the reproductive age group is difficulty in engaging in sexual activity, as was encountered in the present case.^1^ Application of topical estrogen creams is the first choice of treatment in prepubertal girls and postmenopausal women but is not usually successful in the reproductive age group. Consequently, surgical division of the adhesion is a better alternative to medical treatment in women of reproductive age. In the present case, we opted for surgical excision of the adhesion because of its thick fibrous nature.

It has been postulated that low levels of estrogen are a predisposing factor for labial adhesion in prepubertal girls and postmenopausal women.^5^ In one study, labial biopsies obtained from two patients revealed chronic and acute inflammatory changes, indicating that inflammation may play a role in the formation of labial adhesion in hypoestrogenic women.^6^ However, it may also occur in the presence of normal estrogen levels.^7^ In women of reproductive age; female circumcision, herpes simplex, diabetes, pemphigoid, caustic vaginitis, poor hygiene, trauma related to sexual abuse or a fall and lichen sclerosis are reported to be the causes of labial adhesion.^1^,^8^ In the present case, we excluded hypoestrogenemia as a causative factor since the patient’s estrogen levels were normal. She denied a history of trauma, sexual abuse or vaginal discharge. The patient’s history of preeclampsia, repetitive oral ulcers, painless vaginal ulcer and the presence of labial adhesion arouse the suspicion of systemic lupus erythematosus (SLE) as the underlying cause. With the patient’s consent, detailed investigations were performed. In the following months after surgery, 4 of the 11 American College of Rheumatology criteria required for the diagnosis of SLE; ANA positivity, oral ulcers, photosensitivity and immunological disorder presenting with anti-Sm antibody positivity were documented.^9^ It is a rare finding in SLE to have a vaginal ulcerous lesion.^10^ We hypothesized that the asymptomatic vaginal ulcer could have caused a chronic inflammatory process eventually resulting in labial adhesion.

To the best of our knowledge, this is the first case report that shows a relationship between SLE and labial adhesion. We believe that SLE should be considered in the differential diagnosis of labial adhesion in women of reproductive age.

### Authors’ Contribution

**DY and MT** both involved in manuscript writing.
